# Tetrandrine alleviates podocyte injury via calcium-dependent calpain-1 signaling blockade

**DOI:** 10.1186/s12906-021-03469-x

**Published:** 2021-12-14

**Authors:** Yin Ding, Xuanli Tang, Yuhui Wang, Dongrong Yu, Caifeng Zhu, Jin Yu

**Affiliations:** Department of Nephrology (Key laboratory of Management of Kidney Disease in Zhejiang Province), Hangzhou TCM Hospital Affiliated to Zhejiang Chinese Medical University, Tiyuchang Road 453, Hangzhou, 310007 People’s Republic of China

**Keywords:** Tetrandrine, Podocyte injury, Transient receptor potential cation channel protein 6, Calpain-1

## Abstract

**Background:**

Podocytes have become a crucial target for interventions in proteinuric kidney diseases. Many studies have reported that overexpression of transient receptor potential cation channel protein 6 (TRPC6) in podocyte injury upregulates intracellular Ca^2+^ influx and stimulates Ca^2+^-dependent protease calpain-1 signaling. The traditional Chinese drug, tetrandrine, a nonselective Ca^2+^ channel blocker, has long been used to treat chronic kidney disease. This research aimed to explore the possible mechanisms underlying the anti-proteinuric properties of tetrandrine.

**Methods:**

We investigated the involvement of tetrandrine in Ca^2+^ dependent calpain-1 signaling in mouse podocytes and adriamycin-induced nephropathy rats. Cyclosporine A (CsA) and U73122 were used as positive controls. Cell viability, cytotoxicity, Ca^2+^ concentration, calpain activity, and mRNA and protein expression levels of calpain-1 signaling pathways were examined. The clinical and pathological changes were measured.

**Results:**

Tetrandrine decreased intracellular Ca^2+^ influx in cultured TRPC6-overexpressing podocytes. In both in vitro and in vivo studies, the administration of tetrandrine downregulated calpain activity and the expression of calpain-1 and restored the expression of downstream Talin-1 and nephrin. Compared to CsA, tetrandrine treatment exhibited superior inhibitory effects on calpain activity and calpain-1 expression.

**Conclusions:**

Tetrandrine has therapeutic potential in podocyte damage by blocking Ca^2+^-dependent activation of the calpain-1 signaling pathway. Tetrandrine reduced proteinuria, improved renal function, and alleviate renal pathological damage.

**Supplementary Information:**

The online version contains supplementary material available at 10.1186/s12906-021-03469-x.

## Background

Podocytes are a prerequisite for maintaining the selective permeability of the glomerular filtration barrier and glomerular structural integrity. Podocyte injury is typically associated with proteinuric renal diseases, including focal segmental glomerulosclerosis (FSGS), membranous nephropathy, minimal change disease, and diabetic kidney disease [1–4]. Protection and restoration of podocytes from injurious factors constitute a major challenge in the successful treatment of kidney disease.

Podocytes are highly specialized, terminally differentiated cells located outside of the glomerular basement membrane and characterized by foot processes. Dysfunction of calcium signaling regulation in podocytes leads to podocyte actin cytoskeletal disorganization, foot process effacement, disruption of the slit diaphragm, and proteinuria. Transient receptor potential cation channel protein 6 (TRPC6), a novel slit diaphragm-associated protein in podocytes, has been identified as a nonspecific Ca^2+^-conducting ion channel [5–8]. Glomerular TRPC6 expression is increased in many hereditary and acquired proteinuric diseases. Previous studies have reported that overexpressing of TRPC6 in podocytes triggers a superior increase of intracellular Ca^2+^ influx, which is believed to be responsible for podocyte cell injury. Increased calcium load within podocytes activates downstream signaling pathways, including calpain-1, Talin-1, nephrin and calcineurin, to modulate the activity or expression of target genes [9–12].

Fangji Huangqi Tang is a classic traditional Chinese medicine (TCM) for the treatment of nephrotic syndrome [13]. It consists of six herbs: Radix *Stephania Tetrandra* (the roots of *Stephania tetrandra*), Radix Astragali (the roots of *Astragalus membranaceus*), Rhizoma *Atractylodis Macrocephalae* (the roots of *Atractylodes macrocephala*), Radix Glycyrrhizae (the roots of *Glycyrrhiza uralensis*), Rhizoma Zingiberis (the roots of *Zingiber officinale*) and Fructus Ziziphi Jujubae (Fructus of *Ziziphus jujuba*) [1]. Tetrandrine, one of the main active components of Radix *Stephania Tetrandra*, a non-selective Ca^2+^ channel blocker, has been tested in clinical trials and has been indicated to alleviate proteinuria and improve renal function [14, 15]. A previous study demonstrated the blocking effects of tetrandrine on Ca^2+^-dependent RhoA signaling in podocytes [16], but the underlying mechanisms was not elucidated. Herein, TRPC6-overexpressing models were developed in vitro and in vivo. Subsequent to administration of tetrandrine, changes in TRPC6-dependent Ca^2+^ signaling were evaluated, including downstream targets, calpain-1, Talin-1, nephrin and calcineurin. Cyclosporine A (CsA), an inhibitor of calcineurin, widely used in the treatment of proteinuria diseases) [17] and U73122, an inhibitor of TRPC6 channel opening [18] were used as positive controls.

## Methods

### Cell culture and plasmid construction

Conditionally immortalized mouse podocyte cell line (MPC5) cells, obtained from Professor Peter Mundel (Mount Sinai School of Medicine, USA), were maintained at 33 °C for proliferation, and then shifted to 37 °C for differentiation. The TRPC6 expression plasmid was constructed by cloning the full length of the coding DNA sequence of the TRPC6 gene into the EcoRI/BamHI sites of the pCDH-GFP-PURO vector, and packaged into lentivirus. When differentiated and grown to approximately 80% confluence, podocytes were seeded at a cell density of 4 × 10^4^ per cm^2^ and incubated in combined with TRPC6-overexpressing lentivirus. The TRPC6 stable cell line, verified using TRPC6 protein and mRNA expression, was used for further experiments. Thus, MPC5 podocytes were divided into three groups, normal control (NC) group, blank lentivirus (Lv-NC) group, and TRPC6-overexpressing (OverTRP) group.

### In vitro cell drug treatment and cytotoxicity assay

Cell viability was accessed using the Cell Counting kit-8 (CCK-8) assay, according to the manufacturer’s instructions. A total of 5000 MPC5 podocytes per well were grown in triplicate in 96-well plates. Cells were treated with a series of CsA (0–2 μmol/L; Sigma-Aldrich, St. Louis, MO, USA) and tetrandrine (0–40 μmol/L; Sigma-Aldrich) at 37 °C for 48 h. CCK-8 was then added into the plates (10 μL/well), which were then incubated in the dark for 2 h. Absorbance at 450 nm was measured using a microplate reader (BioTek Instruments, USA). Cytotoxicity was assayed by quantifying, lactate dehydrogenase (LDH) release using the Cytotoxicity LDH Assay Kit-WST (Dojindo Laboratories, Japan). As a positive control, cells were treated with 10 μmol/L U73122 (Calbiochem, USA) at 37 °C for 10 min.

### RNA extraction and quantitative reverse transcriptase polymerase chain reaction (qRT-PCR)

Total RNA was prepared from cultured cells and kidney tissues using RNAiso Plus reagent (Takara, Japan) and reverse-transcribed into cDNA PrimeScript™ RT Master Mix kit (Takara) according to the manufactures’s protocol. qRT-PCR was performed using Power SYBR Green PCR Master Mix (Thermo Fisher Scientific, Germany). The primer sequences of target and reference genes are listed in Supplementary Table S1. The data were collected using the ABI Prism 7900HT FAST Real-Time PCR system. Relative quantification of target gene expression was normalized using the comparative 2-ΔΔCt method. Glyceraldehyde 3-phosphate dehydrogenase (GAPDH) was used as the internal control.

### Western blotting

Total protein samples were extracted from cultured cells and kidney tissues. Protein concentrations were determined using a BCA protein assay kit (Thermo Fisher Scientific). Samples were separated on a 10% sodium dodecyl sulfate-polyacrylamide gel and transferred onto a polyvinylidene difluoride membrane. The membrane was then blocked overnight at 4 °C with primary antibodies, namely anti-TRPC6 (Santa Cruz, USA), anti-calpain-1 (Abcam, UK), anti-Talin-1 (Abcam), anti-nephrin (Santa Cruz) and anti- calcineurin (Abcam). After washing, the membrane was incubated with goat anti-rabbit IgG (H + L; Jackson ImmunoResearch Laboratories, USA). The membrane was also processed to detect GAPDH (Abcam) for equal loading. The bands recognized by the primary antibody were visualized, and the optical densities of the protein bands were analyzed using TanonImage software (Tanon, China).

### Determination of cytosolic Ca^2+^ in cultured podocytes

MPC5 podocytes were seeded and treated with tetrandrine, CsA and U73122 for 48 h, 48 h, and 10 min, respectively. Podocytes were then incubated with RPMI-1640 supplemented with 10 μM Fluo-3 AM (Dojindo Laboratories) for 30 min in the dark. Thereafter, they were immediately washed with phosphate-buffered saline, in which they were maintained and excited at 488 nm. The relative concentration of cytosolic Ca^2+^ ion was evaluated by measuring fluorescence intensity using a Zeiss laser confocal imaging system (Zeiss, USA).

### Measurement of calpain activity

Calpain activity was determined using the Calpain Activity Assay Kit from Abcam Inc., according to the manufacturer’s protocol. The supernatant (150 uL) of podocytes or kidney tissues was taken in the appropriate buffer solution (145 mmol/L Nacl, 100 mmol/L Tris-HCL, pH 7.3) and 0.15 mL N-Succinyl-Leu-Tyr-AMC (500 umol/L). After a 1-h incubation at 37 °C in the dark, the samples were read using a fluorometer equipped with a 360 nm excitation filter and a 440 nm emission filter.

### Adriamycin-induced nephropathy (ADRN) rat model

Based on Fermi approximation of sample size [19] and proteinuria difference between the control and model groups in our preliminary experiments, the total number of rats required in our study was determined. A total of 56 healthy male Sprague–Dawley rats, weighing approximately 200 g and aged approximately 8 weeks (Zhejiang Institute of TCM), were randomly divided into seven groups (*n* = 8 rats/group), namely the ADR group, ADR+ tetrandrine low-dose group (4 mg/kg/d), ADR+ tetrandrine middle-dose group (8 mg/kg/d), ADR+ tetrandrine high-dose group (16 mg/kg/d), ADR+ CsA treated group (30 mg/kg/d), ADR+ middle dose of tetrandrine (8 mg/kg/d) in combination with CsA (30 mg/kg/d) group, and NC group. Rats in the model group and drug treatment groups were anesthetized with an intraperitoneal injection of ketamine hydrochloride (50 mg/kg), and two doses of ADR (4 mg/kg) injected into the caudal vein to develop the FSGS rat model. After 12 weeks, urine samples of the rats were collected for 24 h using metabolic cages, followed by collection of blood and harvest of the kidneys. Clinical efficacy was observed, and the changes in 24-h urine protein, plasma albumin, serum creatinine (Scr), and blood urine nitrogen (BUN) levels were measured using commercially available diagnostic kits with an automated chemistry analyzer (HITACHI 7180).

All experimental procedures involving the use of animals were approved by the Ethics Committee on Animal Experiments at Zhejiang Institute of TCM (Tianmushan Road No.132) and were strictly performed according to the principles outlined in the NIH Guide for the Care and Use of Laboratory Animals.

### Histologic staining, electron microscopy and analysis

Rat kidneys were fixed in paraformaldehyde, dehydrated using a graded series of ethanol, and embedded in paraffin. Sections (5-μm-thick) were stained with hematoxylin and eosin (H&E) and Masson’s trichrome and examined under an optical microscope. Kidneys were perfused and fixed with glutaraldehyde and then cut into 1-mm^3^ tissue blocks for epoxy embedding. Electron microscopy was performed using standard procedures. Five ultra-micrographs were randomly obtained from each specimen. Podocyte foot processes fusion rate (PFR) was calculated using the following formula: PFR (%) = ∑LFP / ∑LBM × 100%, where ∑LFP is the total length of the fused foot process and ∑LBM is the total length of the peripheral capillary basement membrane (BM).

### Statistical analysis

Data are expressed as mean ± standard error of measurement and analyzed using SPSS 20.0 (IBM Statistics SPSS, US) and GraphPad Prism 6 for Windows (GraphPad Software Inc., USA). Normal distribution was assessed by qq plots and S-W test. The data showed a normal distribution. When two groups of data were compared, the independent samples t-test was applied. Differences among three or more independent groups were compared using one-way analysis of variance followed by Tukey’s post hoc test. Statistical significance was set at *p* < 0.05.

## Results

### Selection of optimal concentrations and cytotoxicity of tetrandrine, CsA and U73122

**Fig. 1:**
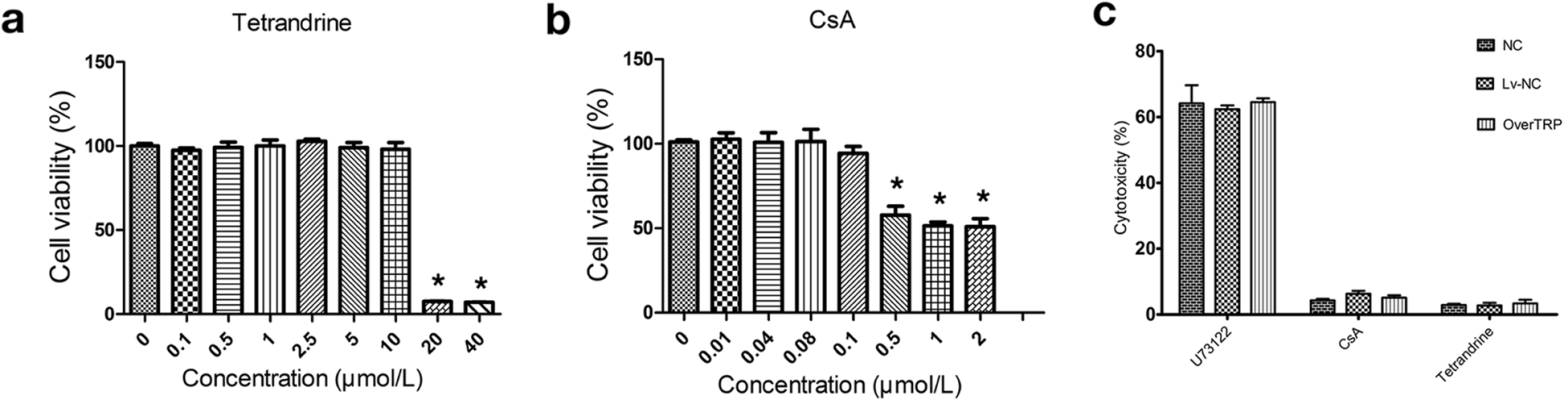
Optimization of drug concentrations. MPC5 podocyte viability under treatment with different concentrations of tetrandrine (**a**) and CsA (**b**) detected by CCK8 assay. **c** The LDH cytotoxicity assay in MPC5 podocytes treated with tetrandrine, CsA and U73122. * P<0.05. NC: normal control. Lv-NC: blank lentivirus. OverTRP: TRPC6-overexpressing

The CCK-8 assay was used to detect cell viability. As shown in the Fig. 1a and b, the maximum concentration of 10 μmol/L of tetrandrine and 0.1 μmol/L of CsA did not affect the viability of MPC5 podocytes. The LDH release assay further confirmed that 10 μmol/L tetrandrine and 0.1 μmol/L CsA for 48 h showed no toxicity, whereas 10 μmol/L U73122 for 10 min showed espected toxicity (Fig. 1c). Therefore, the subsequent cell experiments utilized tetrandrine and CsA at 10 μmol/L and 0.1 μmol/L, respectively.

### Tetrandrine decreased intracellular Ca^2+^ influx in the cultured MPC5 cells overexpressing TRPC6

**Fig. 2:**
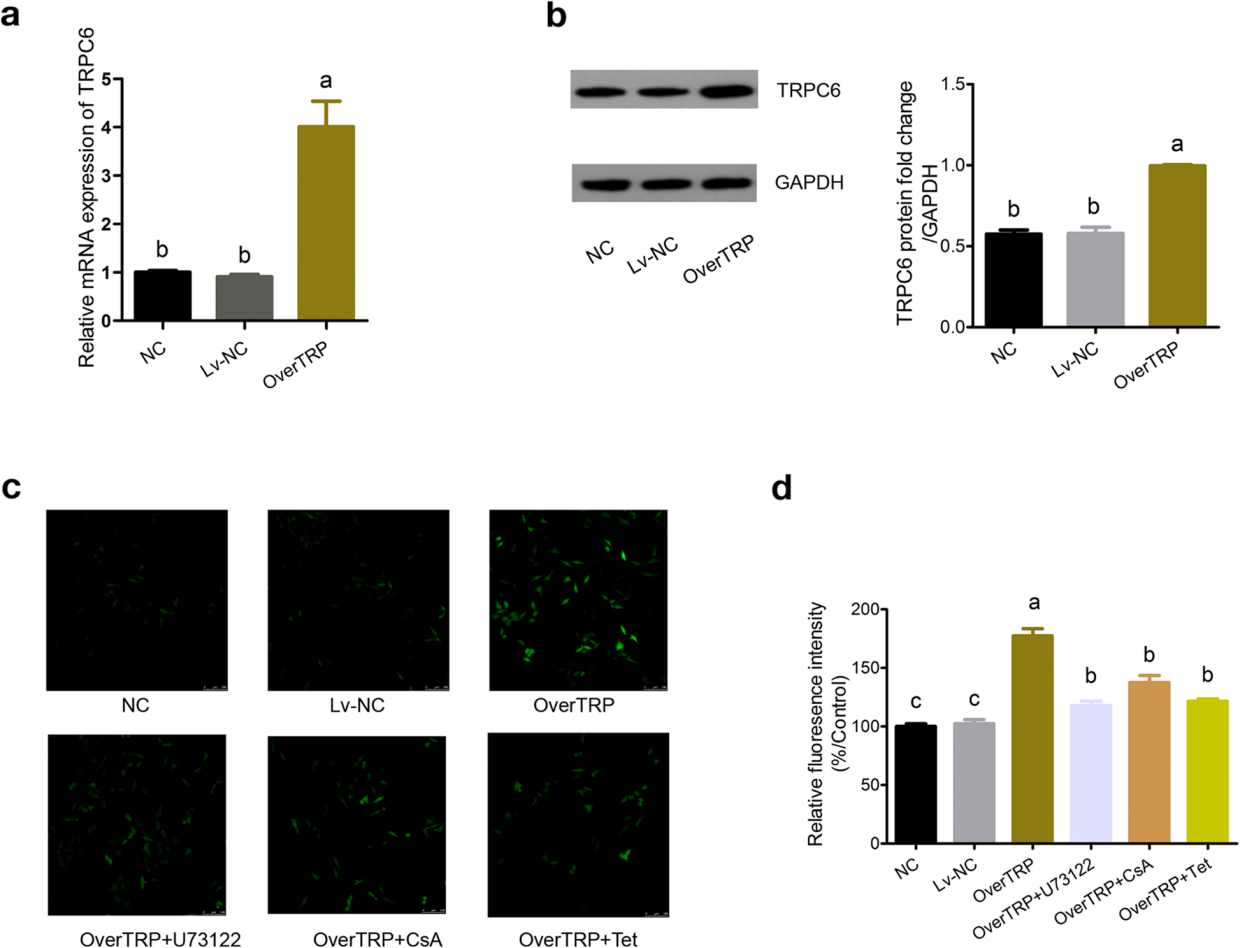
Construction of TRPC6 overexpressing cell model, and detection of Ca^2+^ influx among groups. The mRNA and protein levels of TRPC6 were determined by qRT-PCR (**a**) and western blotting (**b**), respectively. The intracellular Ca2^+^ influx was determined using Fluo-3 AM under confocal microscope (**c**) and semiquantitative analysis (**d**). Different letters represent statistically different values (*P* < 0.05), whereas the same letters indicate no significant difference

Using plasmid transfection, a cell model of TRPC6 overexpression in MPC5 podocytes was successfully established. qRT-PCR and western blot analysis demonstrated that the expression levels of TRPC6 in the OverTRP group increased significantly compared to that in the NC and Lv-NC groups (*p* < 0.05; Fig. 2a and b). And Full-length gel and blot of TRPC6 was included in the Supplementary Fig. S1.

Fluorescence imaging (Fig. 2c) and semi-quantitative analysis (Fig. 2d) indicated that overexpression of TRPC6 enhanced intracellular Ca^2+^ influx, whereas there were no obvious differences between the NC and Lv-NC groups. Then OverTRP group cells were then treated with the experimental drugs. The drug pretreatment groups (tetrandrine, CsA and U73122) had inhibitory effects on intracellular Ca^2+^ influx (*P* < 0.05), while there was no statistically significant difference among the three groups of drugs.

### Effects of tetrandrine on activity and the expression of calpain-1, calcineurin, Talin- 1 and nephrin in MPC5 cells

**Fig. 3:**
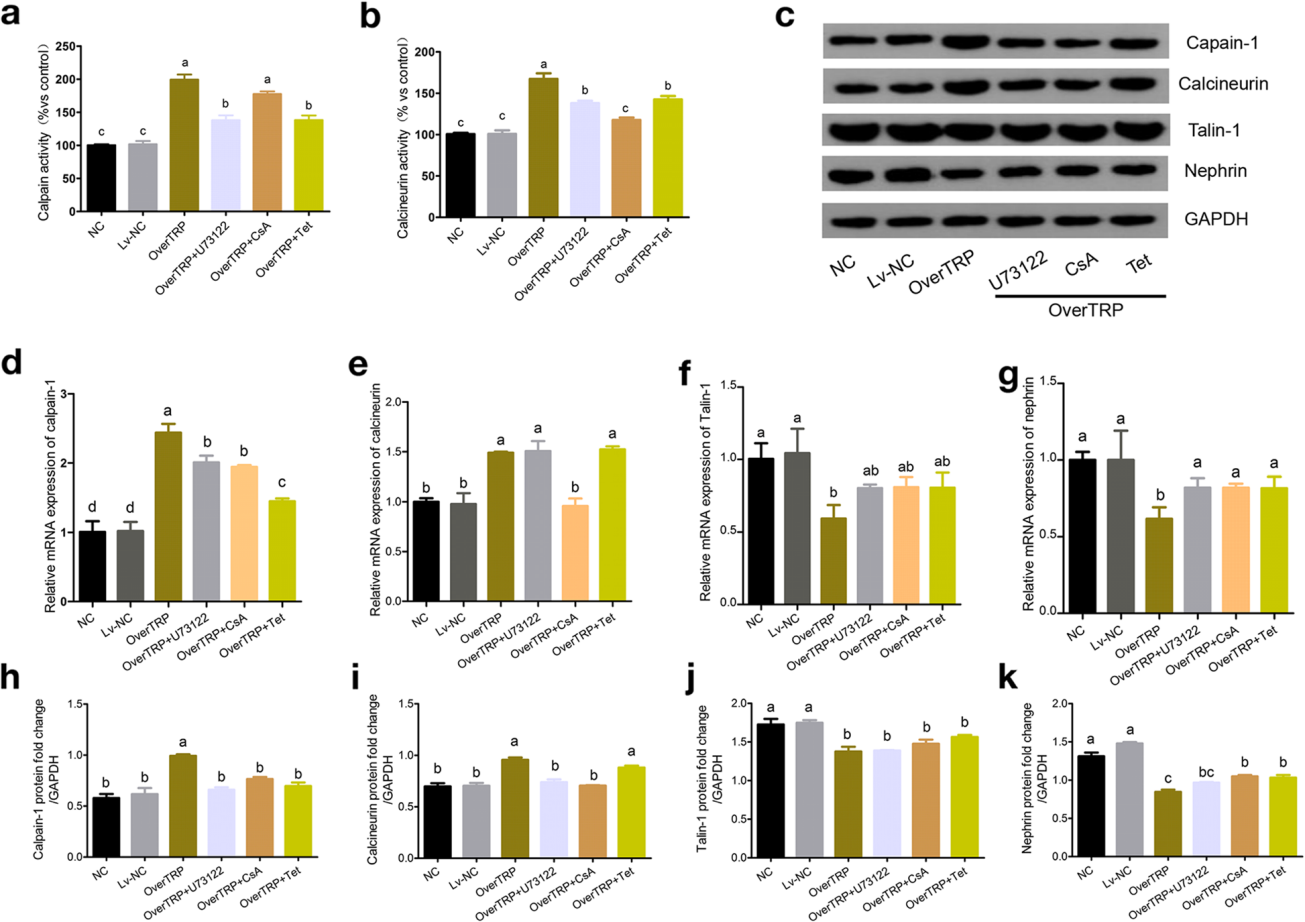
Tetrandrine decreased Ca^2+^-dependent calpain-1 signaling in cultured podocytes. **a** Calpain and **b** calcineurin activity were measured. **c** Typical bands showing the protein levels of TRPC6, calpain-1, calcineurin, Talin-1 and nephrin, normalized to GAPDH. Detection of mRNA **d**-**g** and protein levels **h**-**k** of calpain-1, calcineurin, Talin-1 and nephrin, respectively. CsA and U73122 were used as positive controls. Different letters represent statistically different values (P < 0.05), whereas the same letters indicate no significant difference

It has been shown that intracellular Ca^2+^ activates calpain, a Ca^2+^-dependent cysteine protease, and calcineurin, a Ca^2+^/calmodulin-dependent protein phosphatase. In line with previous observations, the activity and expression levels of calpain and calcineurin in the OverTRP group were all higher than those in the NC and Lv-NC groups (*p* < 0.05; Fig. 3a-e, h and i). Drup intervention in cultured TRPC6-overexpressing podocytes established that tetrandrine inhibited calpain activity and calpain-1 expression. The calpain activity and calpain-1 mRNA levels were significantly reduced in the tetrandrine-exposed group than in the CsA intervention group (*p* < 0.05; Fig. 3a and d). Western blotting results demonstrated the considerable decrease in calpain-1 protein levels because of tetrandrine (*p* < 0.05; Fig. 3c and h). In addition, calcineurin activity was significantly reduced (*p* < 0.05; Fig. 3b) after tetrandrine exposure, but the mRNA and protein expression levels of calcineurin remain unchanged (Fig. 3e and i).

Treatment with CsA did not block calpain activation (Fig. 3a), and the effect was not as robus as that of tetrandrine in calpain-1 mRNA expression (*p* < 0.05; Fig. 3d). In addition, the inhibiting effect on the activity and expression of calcineurin was more obvious in the CsA-treated group than in the tetrandrine-treated group (p < 0.05; Fig. 3b, e and i).

The podocyte cytoskeleton-associated anchoring protein Talin-1 and the slit diaphragm protein nephrin are two specific targets of calpain-1. When treated with tetrandrine, there was an increasing trend in mRNA and protein expression of Talin-1 (*p* > 0.05) and nephrin (*p* < 0.05), compared with that in the OverTRP group, but this finding was not statistically significant among the three treatment groups (p > 0.05; Fig. 3f, g, j, and k).

Full-length gels and blots were included in the Supplementary Fig. S1.

### In vivo experiments using a rat model of ADRN

**Fig. 4:**
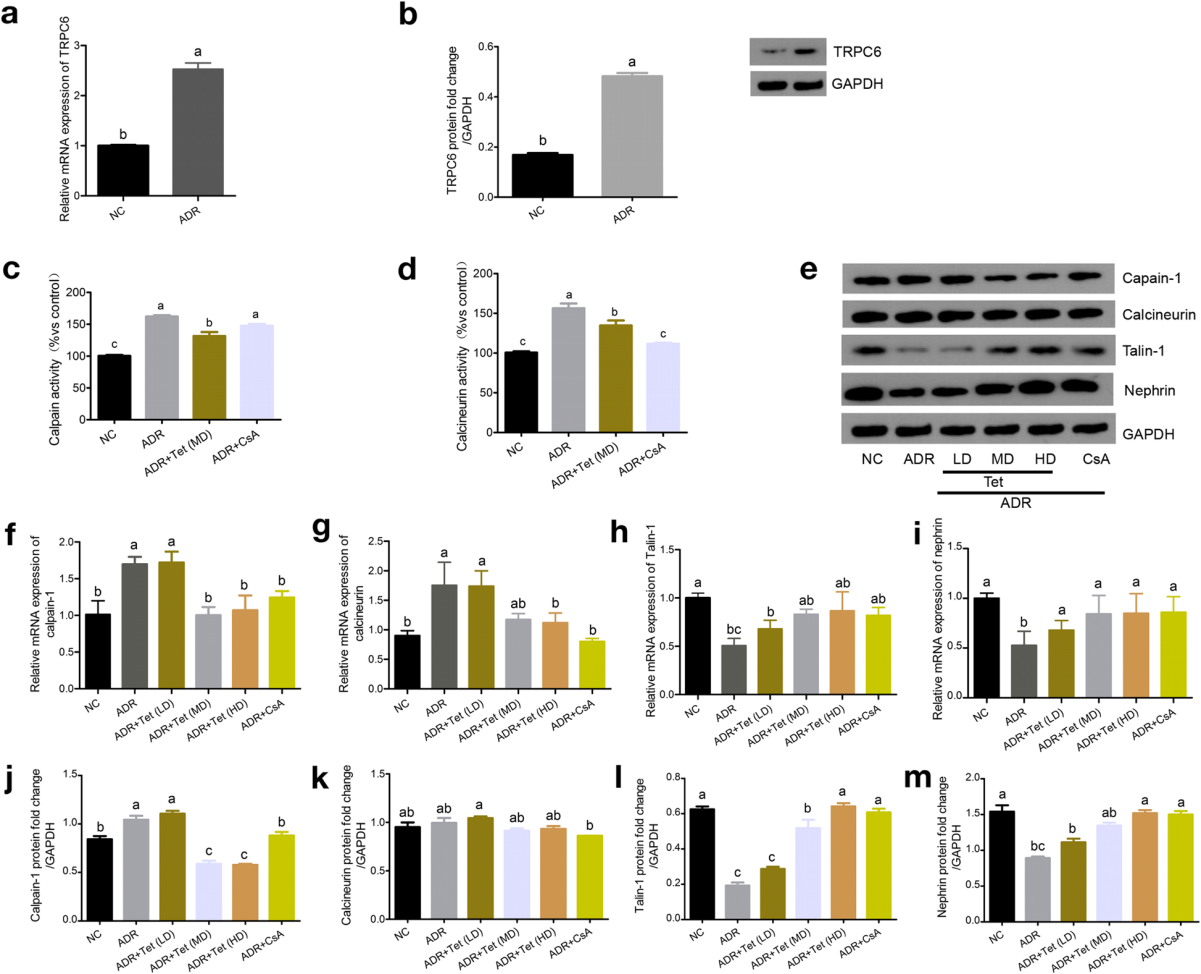
Tetrandrine-mediated inhibition of Ca^2+^/calpain-1 signaling in adriamycin-induced nephropathy rats. TRPC6 overexpressing rat model was established (**a**, **b**). **c** Calpain and (**d**) calcineurin activity was measured. **e** Typical bands showing the protein levels of TRPC6, calpain-1, calcineurin, Talin-1 and nephrin, normalized to GAPDH. The mRNA (f-i) and protein expressions (j-m) of calpain-1, Talin-1, calcineurin and nephrin, respectively. CsA was used as the positive control. Different letters represent statistically different values (P < 0.05), whereas the same letters indicate no significant difference

To characterize the response to tetrandrine-mediated inhibition of TRPC6/calpain-1 signaling in vivo, a model of ADRN was developed in rats. The mRNA and protein expression profiles of TRPC6 were significantly increased in the ADRN group compared to those in the normal-chow fed rats (NC group; *p* < 0.05; Fig. 4a and b). Consistent with the above studies in cell-based models, the activity and expression levels of calpain and calcineurin increased remarkably in ADRN group (p < 0.05; Fig. 4c-g, j and k) than in the NC group.

To determine the effect of tetrandrine, the rats were treated at various concentrations as indicated. A significant decrease in the activity of calpain and calcineurin was observed after tetrandrine exposure, compared with that in the ADR group (*p* < 0.05; Fig. 4c and d). The mRNA and protein levels of calpain-1 declined significantly in the tetrandrine middle- and high-dose groups (*p* < 0.05), whereas their levels were maintained in the low-dose group (*p* > 0.05), compared with those in the ADR group (Fig. 4f and j). The mRNA expression level of calcineurin in the high dose of tetrandrine group was down compared to the ADR group (Fig. 4g).

Treatment with CsA did not block the calpain activation induced by TRPC6 overespression (Fig. 4c), and the effect was not as robus as that of tetrandrine on calpain-1 protein expression (p < 0.05; Fig. 4j). In addition, the inhibiting effect on the activity and expression of calcineurin was more obvious in the CsA-treated group than in the tetrandrine-treated group (p < 0.05; Fig. 4d, g and k).

Furthermore, the expression of Talin-1 and nephrin were evaluated (Fig. 4h, i, l, and m). Both Talin-1 and nephrin levels were reduced in ADRN rats compared to that in the NC group (p < 0.05). Upon treatment with tetrandrine, the mRNA levels and protein expression of Talin-1 and nephrin were visibly increased. The high-dose group of tetrandrine recovered the expression of the two structural components, similar to the ADR + CsA group (*p* > 0.05).

Full-length gels and blots were included in the Supplementary Fig. S2.

### Tetrandrine suppressed ADRN progression and rescued histopathology

**Fig. 5:**
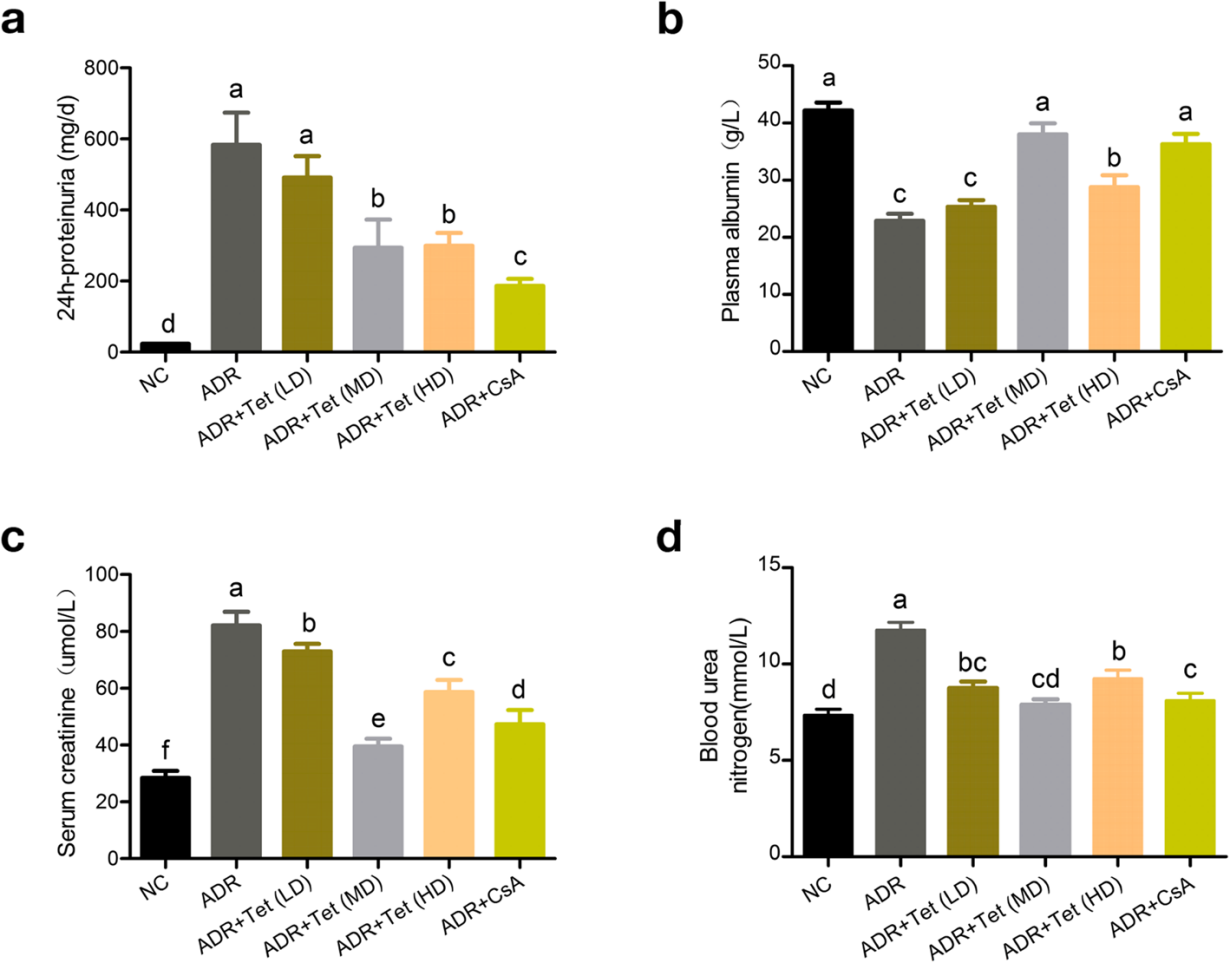
Protective effects of drugs on renal function in adriamycin-induced nephropathy rats. 24 h-proteinuria (**a**), plasma albumin (**b**), serum creatinine (**c**), and blood urea nitrogen (**d**) were measured and compared. Different letters represent statistically different values (P < 0.05), whereas the same letters indicate no significant difference

The effect of tetrandrine on kidney function in Sprague-Dawley rats treated with ADRN was subsequently evaluated (Fig. 5a-d). There were no initial significant differences in 24-h proteinuria, Scr and BUN levels among the groups. Twelve weeks later, the levels of 24 h-proteinuria, Scr and BUN in the ADRN group were statistically increased, whereas that of plasma albumin was markedly reduced, compared to those in the NC group (*P* < 0.05). After treatment with tetrandrine, the levels of proteinuria, Scr and BUN were markedly decreased, whereas the level of albumin was significantly improved compared to those in the ADR group (P < 0.05). Among the three dose-response subgroups of tetrandrine, a significant difference were observed: the middle dose demonstrated considerably better efficacy than that other two doses (P < 0.05). There was no significant difference in the levels of serum alanine aminotransferase, before and after treatment among groups (*P* > 0.05; data not shown).

**Fig. 6:**
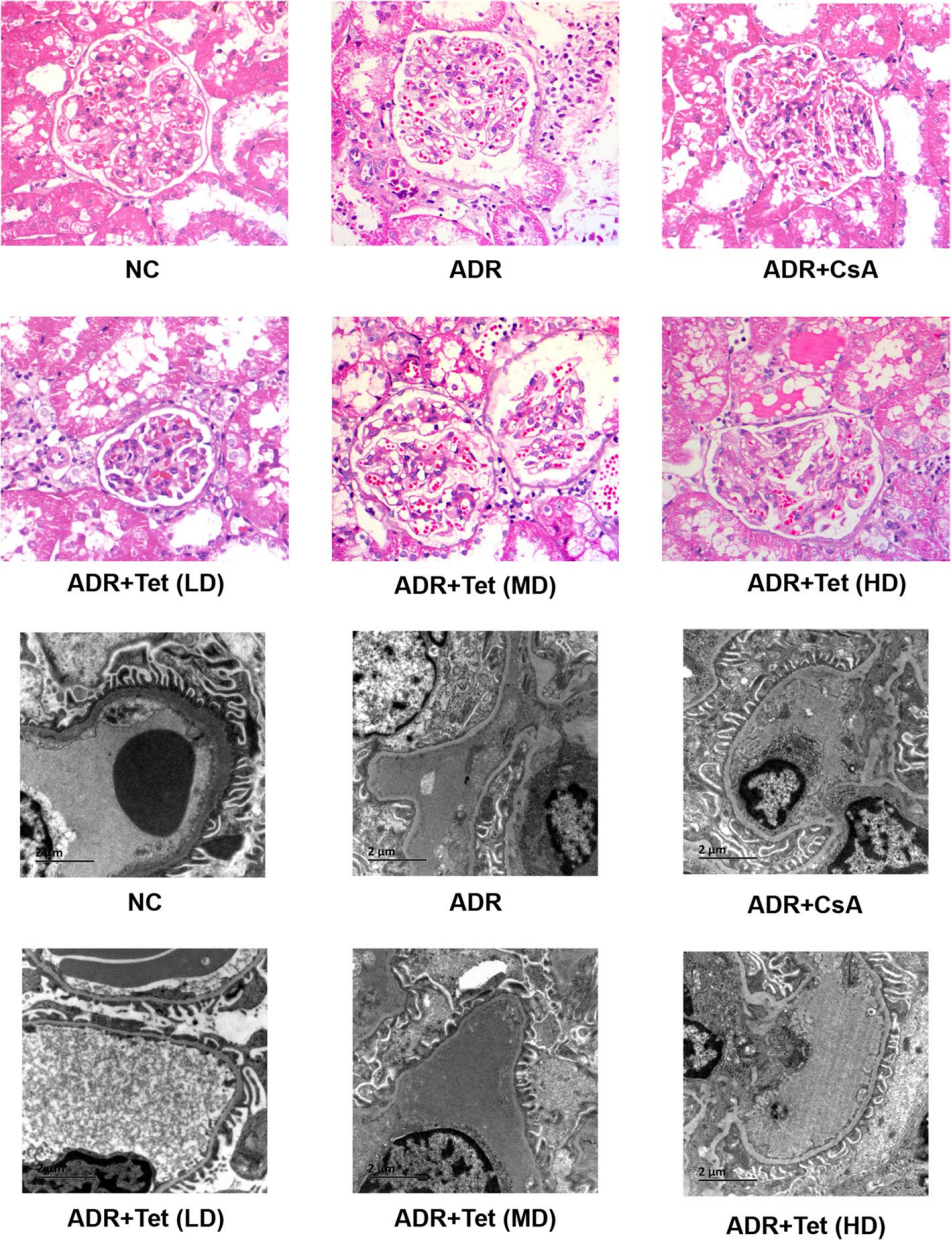
Morphological characteristics of kidney in rats. Typical morphological characteristics of glomerulus under light microscopy (HE staining, magnification: 400×) and electron microscopic evaluation of podocyte structure among different groups of rats (magnification: 20000×)

Changes in histomorphology are shown in Fig. 6. In ADRN rats, H&E and Masson staining showed FSGS, tubulointerstitial fibrosis, and renal interstitial inflammatory cell infiltration. In addition, electron microscopy imaging of glomeruli revealed highly effaced, flattened, and fused podocyte foot processes with 80–100% podocyte PFR. Rats in the tetrandrine sub-groups (middle-dose and high-dose groups) presented with fewer lesions than the ADR group with various extents, and the improvement in the high-dose group excelled that of the middle-dose group. In the low-, middle-, and high- dose of tetrandrine-treated groups, the podocte PFRs were 70 –80%, 50 –60%, and 30 –40%, respectively. These data suggest that tetrandrine protected against structural damage of renal glomeruli induced by ADR administration. There were no significant differences in pathological changes under light microscope among the groups (high-dose tetrandrine, CsA, and tetrandrine in combination with CsA). Additionally, the podocte PFR in the CsA-treated ADRN group was the same as that in the high-dose of tetrandrine-treated groups.

## Discussion

Podocytes have become increasingly crucial as a target for interventions in FSGS and related nephrotic diseases [20, 21]. Herein, based on in vitro and in vivo data, it was demonstrated that the podocyte protection and antiproteinuric effect of tetrandrine, a non-selective Ca^2+^ channel blocker, was mediated via inhibition of intracellular Ca^2+^ influx and Ca^2+^-dependent calpain-1 signaling cascade, including Talin-1, nephrin and calcineurin. Tetrandrine was more effective than in hindering calpain-1 inhibition.

According to previous findings, podocytic TRPC6 is the channel that mainly contributes to changes in Ca^2+^ influx under normal and pathological conditions [7, 22]. It is widely known that calpain activity is dependent on calcium levels and is associated with kidney injury, particularly renal anti-glomerular BM injury [10, 23]. Among the 15 calpain isoforms identified, calpain-1 has recently been shown to link TRPC6 activity to podocyte injury [24]. Calpain-1 is reported to cleave the podocyte cytoskeleton–associated anchoring protein Talin-1 and the slit diaphragm protein, nephrin, which disturbs podocyte integrity and induce severe proteinuria [8–12, 16]. Calcineurin is a Ca^2+^-dependent phosphatase that has previously been demonstrated to play a role in podocytes and kidney function. Accumulating evidence increasingly indicates that calpain can cleave and activate calcineurin in a proteolytic manner [25, 26]. This study created TRPC6 overexpression models by virus infection in cultured podocytes and adriamycin injection in SD rats. In accordance, we showed that TRPC6-medicated Ca^2+^ influx activated the calpain activity and the calpain family member calpain-1 expression. Increased calpain-1 subsequently cleaved the downstream Talin-1 and nephrin.

Tetrandrine is an alkaloid extracted from a traditional China medicine plant and is a non-selective Ca^2+^ channel blocker. Herein, it was reaffirmed that tetrandrine inhibited TRPC6 overexpression and reduced the influx of Ca^2+^ in both cells and rats overexpressing TRPC6, as documented previously [16]. More importantly, this study focus on the intervening effect of tetrandrine on the calcium-dependent downstream signaling pathway. It was observed that unlike CsA, tetrandrine reduced calcium-activated neutral protease (calpain) activity. Furthermore, tetrandrine decreased the expression of the calcium-activated protease family, calpain-1, and restored the downregulated expression of Talin-1 and nephrin. In comparison with CsA, tetrandrine administration exhibited superior effects on calpain inactivation and downregulated expression of calpain-1. These results showed that tetrandrine has therapeutic potential in podocyte damage by blocking TRPC6-dependent activation of the calpain-1 signaling pathway.

Phospholipase C (PLC) has been identified to be a TRPC6-binding partner [27]. U73122 inhibits PLC, so that less diacylglycerol is generated and the TRPC6 channel opening is reduced [18, 24]. U73122 was, thus, used in parallel as a positive control, and it did show a noticeable effect in reducing intracellular Ca^2+^ influx, inhibiting calpain activity and calpain-1 expression in podocytes overexpressing TRPC6. However, in vitro cytotoxicity assays showed apparent podocyte toxicity.

In this study, after 12 weeks of ADR injection, serious proteinuria, hypoalbuminemia, and elevated BUN and Scr levels were evident in ADR--treated rats. Light microscopy revealed FSGS, tubulointerstitial fibrosis, and renal interstitial inflammatory cell infiltration in the ADRN rats. Electron microscopy demonstrated widespread effacements of foot processes. The in vivo data further demonstrated that tetrandrine therapy can reduce proteinuria, improve renal function, and attenuate the progression of glomerulosclerosis. After tetrandrine treatment, pathological changes under light microscope and electron microscope, 24-h proteinuria, plasma albumin, Scr and BUN in ADRN rats were significantly improved compared to those in the NC group.

The calcineurin inhibitor, CsA, has long been used to treat proteinuric kidney diseases [28]. Calcineurin is clinically important as a direct target of the immunosuppressive drug CsA [17, 29]. CsA reduces the expression of calcineurin and diminished calcineurin-mediated nuclear factor of activated T-cells [30–32]. Recent evidence supports the stabilizing effect of CsA on the podocyte actin cytoskeleton, such as synaptopodin by blocking the calcineurin-mediated dephosphorylation of synaptopodin [17]. It has also been demonstrated that calpain can cleave and activate calcineurin in a proteolytic manner [25, 26]. Data from this study reaffirmed that CsA inhibited calcineurin activity and expression more strongly than tetrandrine. As previously reported, CsA does not appear to directly affect calpain activity.

A major limitation of the study was the failure to demonstrate a dose-dependent effect among the tetrandrine sub-groups (low-dose, middle-dose, and high-dose groups). One possible explanation could be that the dose range we used was too high for rats. In addition, the individual differences in the animal experiment may be considerably greater than the dose differences. The relatively small sample size may reduce the statistical power. Therefore, further studies including considerably smaller doses and appropriate sample size are needed to ascertain the exact dose range and effect of tetrandrine in ARDN rats.

In summary, in the regulation of calcium signaling pathways, tetrandrine exhibited an obvious inhibitory effect on calpain-1, whereas CsA directly influenced calcineurin inhibition. These findings suggest that combined tetrandrine and CsA treatment may affect multiple targets of the calcium signaling network to improve clinical outcomes. Confirmatory studies are necessary to validate these observations as well as to elucidate the mechanisms of synergistic effects.

## Conclusion

In conclusion, this study provides evidence that tetrandrine protects podocytes by inhibiting Ca^2+^ influx and blocking calcium-dependent protease calpain-1 to cleave Talin-1 and nephrin, both in TRPC6-overexpressing podocytes and in a rat model of ADRN. Tetrandrine is reconfirmed to reduce proteinuria, improve renal function and alleviate renal pathological damage in animal experiments. Taken together, these results suggest that tetrandrine plays an important role in protecting against podocyte injury.

## Supplementary Information

**Additional file 1: Supplementary Table S1**. Sequences of the primers used in this study.

**Additional file 2: Supplementary Figure S1**. Protein expressions determined by western blotting in cultured podocytes.

**Additional file 3: Supplementary Figure S2**. Protein expressions determined by western blotting in adriamycin-induced nephropathy rats.

## Data Availability

The datasets used and/or analyzed during the current study are contained within the manuscript and the appendix, and available from the corresponding author on reasonable request.

## Abbreviations

FSGS: Focal segmental glomurular sclerosis
MCD: Minimal change disease
MN: Membranous nephropathy
DKD: Diabetic kidney disease
TRPC6: Transient receptor potential cation channel protein 6
Ca^2+^: Calcium
TCM: Classic traditional Chinese medicine
Tet: Tetrandrine
CsA: Cyclosporine A
Scr: Serum creatinine
BUN: Blood urine nitrogen
PFR: Processes fusion rate
BM: Basement membrane
ADRN: Adriamycin-induced nephropathy
PLC: Phospholipase C
