# Supplementary material for: Tetrandrine alleviates podocyte injury via calcium-dependent calpain-1 signaling blockade
Source: BMC Complement Med Ther. 2021 Dec 14;21:296. doi: 10.1186/s12906-021-03469-x (PMC8670271; doi:10.1186/s12906-021-03469-x)
Supplement: Supplementary file 1 — Additional file 1: Supplementary Table S1. Sequences of the primers used in this study. [file 12906_2021_3469_MOESM1_ESM.docx]

| Gene | Primer (5’→3’) |
| --- | --- |
| TRPC6 | F:GCAGCTGTTCAGGATGAAAAC  R:TTCAGCCCATATCATGCCTA |
| Calpain-1 | F:CACCAAGGAAGCCAGCCCCAG  R:GTTTTCATGGCGGCCCAAGCC |
| Talin-1 | F:GGAAATCTGCCGGAGTTTGG  R:TTGGCTGTTGGGGTCAGAGA |
| Nephrin | F:CCCCAACATCGACTTCACTT  R:GGCAGGAVATCCRTGAG |
| GAPDH | F：GGTGAAGGTCGGTGTGAACG  R：CTCGCTCCTGGAAGATGGTG |
