# Supplementary figures and images for: Tetrandrine alleviates podocyte injury via calcium-dependent calpain-1 signaling blockade
Source: BMC Complement Med Ther. 2021 Dec 14;21:296. doi: 10.1186/s12906-021-03469-x (PMC8670271; doi:10.1186/s12906-021-03469-x)

TRPC6


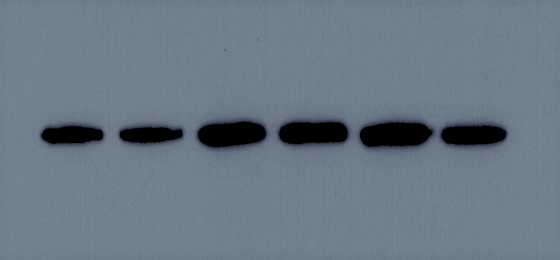


Calpain-1


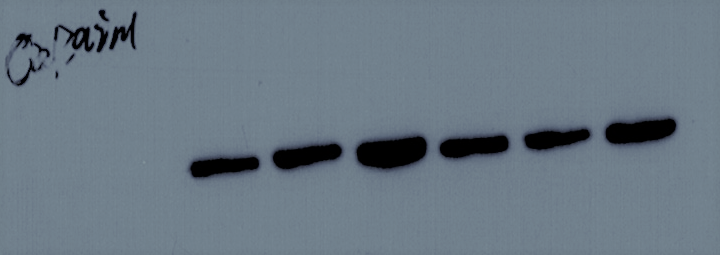


Calcineurin


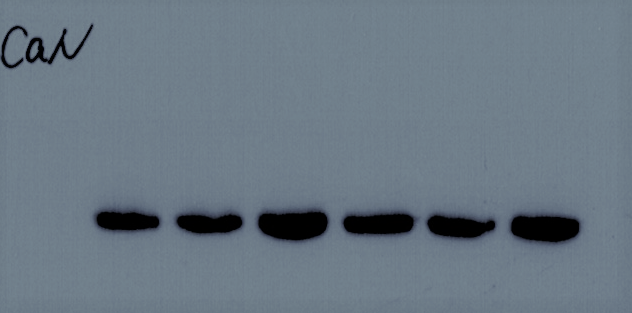


Talin-1


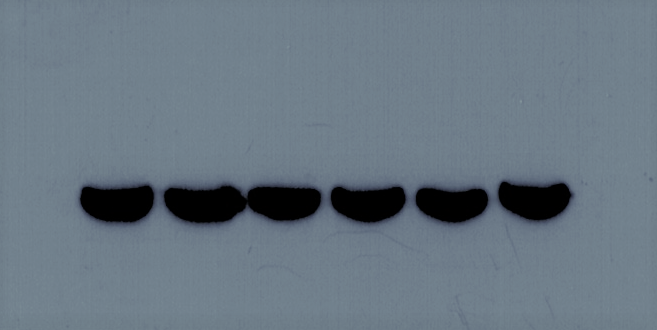


Nephrin


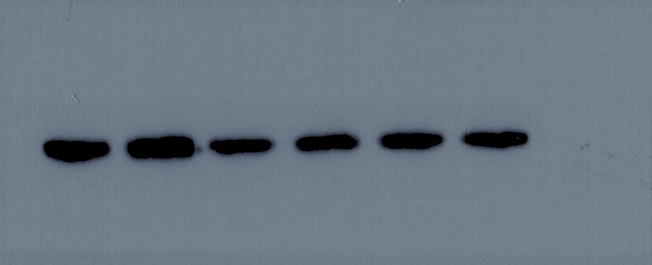


GAPDH


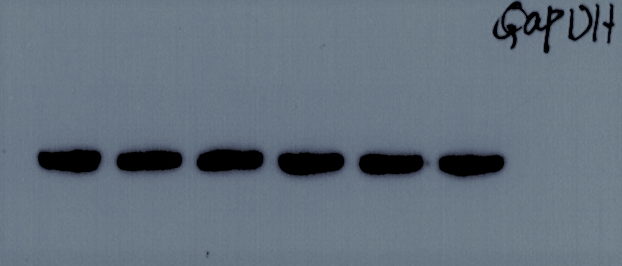

Supplement: Supplementary file 2 — Additional file 2: Supplementary Figure S1. Protein expressions determined by western blotting in cultured podocytes. [file 12906_2021_3469_MOESM2_ESM.docx]

TRPC6


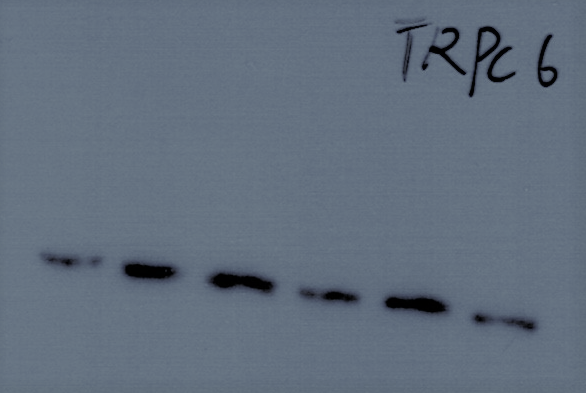


Calpain-1


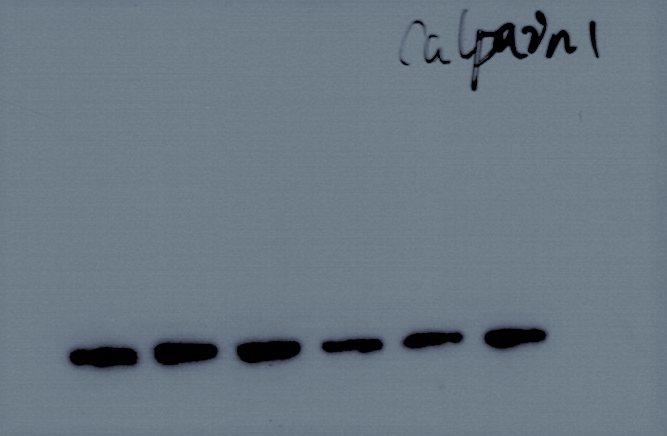


Calcineurin


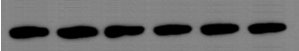


Talin-1


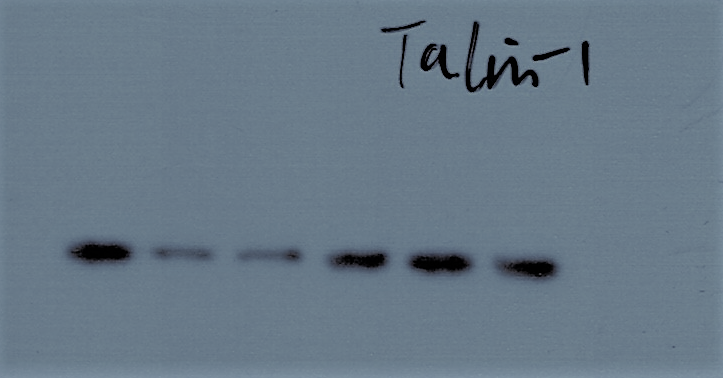


Nephrin


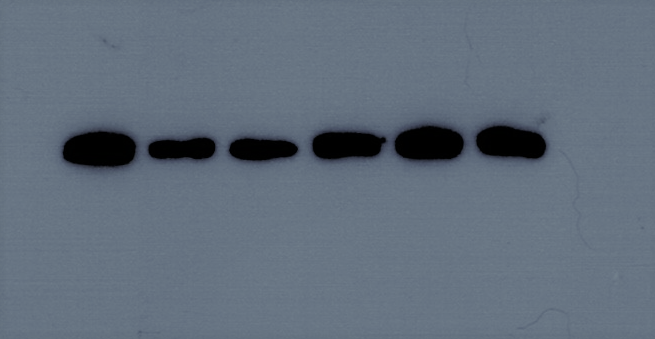


GAPDH


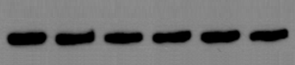

Supplement: Supplementary file 3 — Additional file 3: Supplementary Figure S2. Protein expressions determined by western blotting in adriamycin-induced nephropathy rats. [file 12906_2021_3469_MOESM3_ESM.docx]
